# Scale-Aware Multi-View Reconstruction Using an Active Triple-Camera System

**DOI:** 10.3390/s20236726

**Published:** 2020-11-25

**Authors:** Hang Luo, Christian Pape, Eduard Reithmeier

**Affiliations:** Institute of Measurement and Automatic Control, Faculty of Mechanical Engineering, Leibniz University Hannover, Nienburger Str. 17, 30167 Hannover, Germany; Christian.Pape@imr.uni-hannover.de (C.P.); sekretariat@imr.uni-hannover.de (E.R.)

**Keywords:** active triple-camera measurement system, parallel surface refinement, camera pose estimation

## Abstract

This paper presents an active wide-baseline triple-camera measurement system designed especially for 3D modeling in general outdoor environments, as well as a novel parallel surface refinement algorithm within the multi-view stereo (MVS) framework. Firstly, the pre-processing module converts the synchronized raw triple images from one single-shot acquisition of our setup to aligned RGB-Depth frames, which are then used for camera pose estimation using iterative closest point (ICP) and RANSAC perspective-n-point (PnP) approaches. Afterwards, an efficient dense reconstruction method, mostly implemented on the GPU in a grid manner, takes the raw depth data as input and optimizes the per-pixel depth values based on the multi-view photographic evidence, surface curvature and depth priors. Through a basic fusion scheme, an accurate and complete 3D model can be obtained from these enhanced depth maps. For a comprehensive test, the proposed MVS implementation is evaluated on benchmark and synthetic datasets, and a real-world reconstruction experiment is also conducted using our measurement system in an outdoor scenario. The results demonstrate that (1) our MVS method achieves very competitive performance in terms of modeling accuracy, surface completeness and noise reduction, given an input coarse geometry; and (2) despite some limitations, our triple-camera setup in combination with the proposed reconstruction routine, can be applied to some practical 3D modeling tasks operated in outdoor environments where conventional stereo or depth senors would normally suffer.

## 1. Introduction

Accurate 3D reconstruction is in high demand for a wide variety of tasks such as digital documentation, reverse engineering and industrial inspection. The traditional modeling approaches are mainly categorized into binocular stereo, structured light sensing, and time-of-flight (ToF) measurement. High-end ToF laser scanning systems and structured light sensors have been widely deployed for industrial applications where high modeling precision needs to be guaranteed. Although most laser scanners are capable of directly delivering dense point clouds with a high accuracy and can be applied in outdoor environments, they are normally expensive, time-consuming for data acquisition, and require an additional registration process to add texture information. Similar to laser scanners, industrial structured light sensors (e.g., GOM ATOS CORE 3D Scanner) can produce accurate and traceable 3D coordinates as well as high resolution polygon meshes for surface inspection, 3D printing, etc. However, such systems are also costly and measurements are mostly operated in a controlled indoor environment.

With the development of computer vision and semiconductor technology, consumer grade depth sensors based on structured light and ToF techniques have become available, such as the structured light based Kinect v1 and ToF based Kinect v2. The biggest advantage of these commodity sensors is that they are able to capture the color and aligned depth data per pixel at a real-time frame rate, which makes them even more popular than those high-precision but costly and cumbersome scanning systems in those applications (like robotic navigation and human-machine interaction ) where data acquisition rate is more important than data quality. Even though their application for dense mapping and 3D modeling can be found in a vast literature, e.g., [1,2,3,4,5,6], their inherent characteristics such as noisy, low-resolution depth measurements and wide field of view (FOV) of color camera, prevent them from being solely used for high-quality 3D object modeling tasks. For example, Izadi et al. [1] utilize a volumetric integration approach based on the truncated signed distance function (TSDF) to fuse raw noisy depth maps from a Kinect sensor, yielding a smooth 3D model of an indoor tabletop scene. Note that even though raw depth noise is well suppressed in their results, fine-scale scene structures are heavily smoothed due to the weighted averaging operations in volumetric integration. On basis of this work, Steinbruecker et al. [5] and Nießner et al. [6] adopt more efficient TSDF-encoded structures to reduce memory consumption and improve computational efficiency, but gain only marginal enhancement in surface quality.

Since most depth sensors additionally provide color images at a much higher resolution than that of depth images, researchers thereby have proposed various approaches to enhance the depth measurements by leveraging visual cues in color images. One straightforward idea is to raise the low-resolution depth images to the high resolution of the color image through joint bilateral upsampling and its variants [7], on basis of the assumption about the co-occurrence of intensity and depth discontinuities. While this strategy is fast and the output model might be visually pleasant, the metric accuracy of the reconstructed fine-scaled structures cannot be promised. Wu et al. [8] use shading information in color images to enhance raw depth data from structured light depth sensors, and allows for the real-time refinement based on a GPU-based parallel Gauss-Newton solver. The work in [9] focus on one-shot fusion of ToF measurements and basic pairwise stereo evidences within a probabilistic framework. Kim et al. [10] proposes a multi-view fusion approach utilizing an experimental setup composed of multiple ToF sensors and color cameras for detailed and complete modeling. While these aforementioned works achieve impressive improvement on depth quality, it is worth noting that the data acquisition of these experiments are mostly conducted at a close range in indoor scenarios, in order to improve the quality of raw depth data and in the meantime obtain sufficient spatial resolution for the objects in color images.

Regarding stereo techniques, traditional binocular stereo vision is typically referred to as a passive stereo system composed of two cameras, which estimates the pixel depth by matching and triangulating correspondences across two views. Basic stereo setup can be built on a small budget, and compared with other techniques its data acquisition is much faster and more convenient. The downsides are relatively low depth quality and heavy dependence on texture information due to the matching process. To overcome these limitations, active stereo systems have been proposed to improve the modeling accuracy and completeness by enriching textures in the scenes using an external projection system. Similar to its structured light and ToF counterparts, compact commodity RGB-D cameras based on active stereo vision are developed, such as Intel RealSense D435 composed of a pair of IR cameras, an infrared projector as well as a color camera, enabling real-time acquisition of color and depth data at a moderate resolution but suffering from limited triangulation accuracy due to the short baseline. Although there exist wide-baseline active stereo systems (like GOM ARAMIS 3D Camera series) specially designed for high-precision industrial inspection, most of them are not suitable for outdoor 3D modeling tasks either.

Most modeling processes using active stereo systems are operated in a traditional range scanning manner, where segments of point cloud are individually retrieved from static stereo images at every acquisition and are further merged into a dense cloud via geometric registration [11,12]. For these tasks, the modeling accuracy is mainly determined by the pairwise stereo reconstruction, as well as the final registration process. In the context of active stereo, few papers investigate how to refine single-shot measurement using image information acquired from different views, namely the core idea of MVS reconstruction. This is because the embedded projector in an active stereo system changes the appearance of objects when the device is moved around, which cannot satisfy the conditions of standard MVS pipeline [13] where obvious appearance variations are not allowed during the whole data acquisition process. Harvent et al. [14] presents a dense 3D modeling approach using multiple stereo image pairs captured by an active stereo system at different poses. To refine coarse depths from static stereo, they reformulate the pixel-wise energy function by accumulating pairwise photo differences of multiple stereo pairs linked through local homographies. Their implementation does not solve the problem of changing appearance, and thereby cannot fully utilize the constraints between images from different stereo pairs.

In this paper, we propose a novel multi-view dense reconstruction system that is capable of achieving detailed and complete 3D modeling of objects even in general outdoor scenarios. Specifically, the main contributions of our work are (1) an active triple-camera measurement setup (it is designed to be mounted on a hexacopter that does not allow short-range captures due to the aircraft size and flight safety) that enables the synchronized acquisition of depth data as well as the original texture information of objects, and (2) a highly parallel surface refinement algorithm that improves coarse depth quality on the basis of multi-view photographic information, surface curvature, and depth priors. The use of stereo depth in our system implicitly recovers the metric scale of objects that cannot be obtained from other pure image-based MVS methods.

This paper is arranged as follows. In Section 2 a detailed introduction of the designed measurement setup is presented. Section 3 describes the specific implementation of our proposed reconstruction routine. Afterwards, the results on synthetic and experimental data are demonstrated in Section 4 and further discussed in Section 5. A short summary of our work is given in Section 6, and in the end a table of abbreviations used in this paper is provided.

## 2. Experimental Setup and Data Acquisition

In this section, we firstly give an overview of our triple-camera measurement system, and then describe how to perform synchronized image acquisition using this system.

### 2.1. Active Triple-Camera Measurement Setup

**Figure 1:**
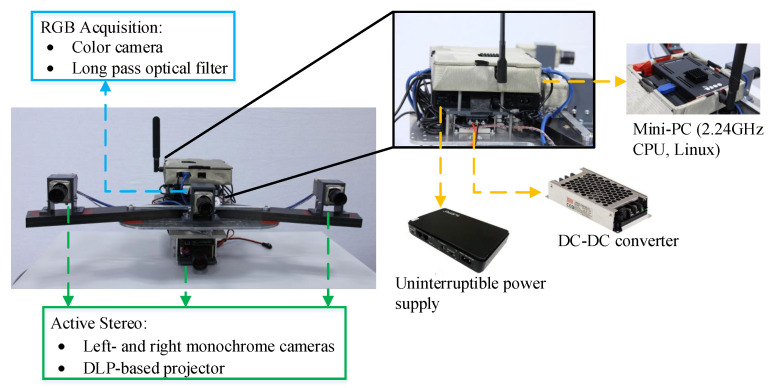
Active triple-camera experimental setup. Left- and right monochrome cameras with the projector constitute an active stereo system, and the middle color camera captures the object textures with a long pass optical filter (useful range: 510–1100 nm).

Figure 1 illustrates the prototype of the setup, which consists mainly of two monochrome cameras (Basler acA1300-200um, 1280 × 1024p) performing pairwise stereo, one color camera (Basler acA1440-220uc, 1440 × 1080p) for the acquiring texture information and a high-brightness programmable LED projector (Wintech LightCrafter PRO4500, 912 × 1140p DMD) to project random patterns for stereo vision as well as to synchronize image captures of these three cameras. In addition, a mini-PC (UDOO X86 ADVANCED) along with its power supply is employed to communicate with the client PC and control the data acquisition process that is described in the following section, and a DC-DC converter is equipped to convert the output voltage from our hexacopter to power the projector. The total weight of this setup is about 5.2 kg. Note that in this paper we only present the results of hand-held experiments conducted using the proposed setup, and therefore the power of the projector is also provided by a power bank instead of this converter during our experiment.

As shown in Figure 1, all these components are mounted on an aluminum base board and we extend the stereo baseline using carbon fiber tubes due to their high stiffness and light weight. Since we focus on detailed dense modeling, relatively long-focus optical lenses are equipped with the camera sensors (16 mm for stereo cameras and 12 mm for the color camera) to raise the spatial resolution of targeted objects in captured images, which is beneficial to the subsequent MVS reconstruction process. This pair of stereo cameras with the color camera are synchronized via the hardware trigger signal that comes from the projector when it starts to display patterns. In order to obtain the original appearance of objects, a long pass filter that blocks virtually all blue light and passes longer visible wavelengths, is attached to the lens of the color camera to remove projected patterns as far as possible while the LED projector is configured to operate on its blue light engine.

### 2.2. Image Acquisition

**Figure 2:**
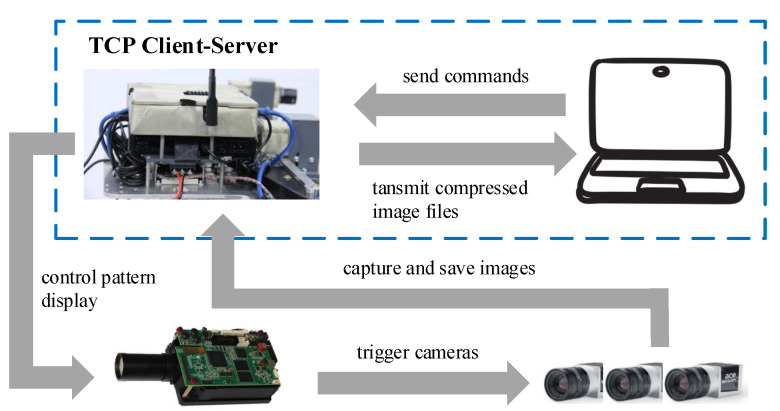
Schematic of image acquisition process. The laptop and the mini-PC constitute a simple client-server architecture using TCP socket programming, thereby enabling the operators to start, pause and stop the synchronous acquisition by pressing corresponding keys. The compressed color image is transmitted back to the laptop for display, based on which the pose of the setup can be adjusted to achieve a better view of ROI.

In our experiment we use a laptop to communicate with the mini-PC through the local wireless network, thereby controlling the image acquisition of the sensors. The complete workflow is depicted in Figure 2, where the projector is configured in software trigger mode and cameras are in hardware trigger mode using their respective SDK tools. To start the acquisition, the laptop sends a command (encoded with predefined key-presses) to the mini-PC via the established TCP connection. Based on the specific command, the mini-PC controls the projector to display patterns in the corresponding modes (The projector has two different display modes: one-time display and continuous display. When operated in one-time display mode the projector display pattern only once after receiving the command, while in continuous mode the pattern is projected repeatably following a pre-set period), and each display action would trigger all cameras to synchronously conduct one-shot capture. The captured images are directly saved on the mini-PC for subsequent off-line reconstruction tasks, and in the meantime transmitted to the laptop in a compressed format for (nearly) real-time view if required.

**Figure 3:**
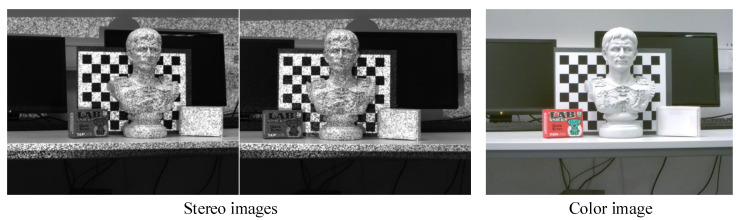
Synchronized triple images from one-shot acquisition. From left to right are respectively stereo gray image pair with active blue random patterns and the color image after long pass filtering.

Figure 3 shows an example of one triple-images pair synchronously captured in our laboratory at a distance of approximately 2 m, and from the color image we can see that the projected blue patterns are well suppressed through the optical filter.

## 3. Method

The main modules of our proposed dense reconstruction pipeline are detailed in this section. At first we briefly introduce the triple-camera calibration along with the preparation work for raw images. Afterwards, the utilized pose estimation approach that sequentially combines multi-way iterative closest point (ICP) registration [15] and RANSAC perspective-n-point (PnP) tracking [16] are described, which is specially designed for our use cases. In the end, a multi-view reconstruction algorithm as well as its implementation on a consumer-grade GPU is elucidated.

### 3.1. Camera Calibration and Data Preprocessing

Since our system bases on active stereo rather than structured light technique utilizing camera-projector correspondences, we only need to calibrate camera intrinsic parameters along with the rigid transformations between left-right cameras for stereo depth estimation and left-color cameras for the alignment of depth and color frames. For such a wide baseline setup, the camera intrinsics are at first independently calibrated using the standard chessboard method [17], and afterwards with the fixed intrinsic matrix the 6-DoF relative transformations between camera pairs are estimated from pairwise chessboard images as well. The utilized chessboard has 10 columns and 7 rows with a square size of 35 mm, and the calibration software is implemented using OpenCV library [18]. Table 1 lists the estimated camera intrinsic and extrinsic parameters used in the following experiments.

**Figure 4:**
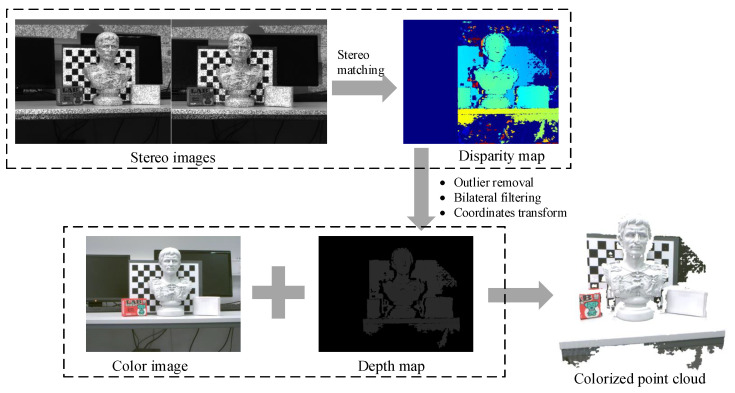
Data preprocessing is performed to convert raw triple images including stereo pairs and the color image into aligned RGB-Depth frames.

In order to obtain aligned depth and color frames, a set of operations are executed on raw image pairs, as depicted in Figure 4. Firstly, semi-global block matching [19] is applied to compute disparity values for the (rectified) left image. Afterwards, 3D point coordinates converted from the disparity map are transformed and projected onto the color frame to generate an aligned depth image, followed by general outlier removal and joint bilateral filtering. The projected colorized point cloud suggests that the registered depth data matches well with the corresponding color pixels. The aligned RGB-D data is subsequently fed to pose estimation and dense reconstruction modules.

### 3.2. Pose Estimation

In a standard MVS framework, accurate pose estimation is of great importance to the dense reconstruction process, which requires relative transformations to compute and aggregate photo costs across neighboring views for depth optimization in the reference view. In the context of RGB-D sensors, ICP along with its variants [20,21] that minimize the Euclidean distances between 3D-3D correspondences, has proved to be effective when registering two coarsely-aligned 3D point clouds. However, its registration accuracy is limited by the uncertainty in raw depth measurements, and outliers of data might lead to incorrect alignments. In our case, the setup is designed to be applied in outdoor scenarios where ambient lighting and environments tend to introduce outliers and artifacts in the disparity computation, and in addition a long working distance (approx.2m in our experiment) increases the uncertainty of triangulated depth estimates, resulting in a relatively poor positioning accuracy. Therefore, the RANSAC PnP in combination with nonlinear pose optimization [22] utilizing 3D points and their corresponding 2D projections in images is exploited to further refine the ICP estimations, as 3D-2D correspondences are generally more robust than 3D-3D associations in particular when encountering input data contaminated by outliers and noise.

With regard to the specific implementation, the multiway ICP registration approach in [23] is utilized for the coarse alignment of point cloud segments from RGB-D pairs. It performs pose graph optimization based on line processes to improve registration accuracy in a global space. Next we feed the RGB-D images along with the coarse pose estimates to a modified ORB-SLAM2 library [22] that performs RANSAC PnP tracking followed by motion-only bundle adjustment, and in the end the integrated pose graph optimization using pose-pose constraints and global bundle adjustment in [22] are invoked in succession to jointly adjust global camera poses and sparse structures. The results before and after refinement are demonstrated in Section 4.

### 3.3. MVS Dense Reconstruction

**Figure 5:**
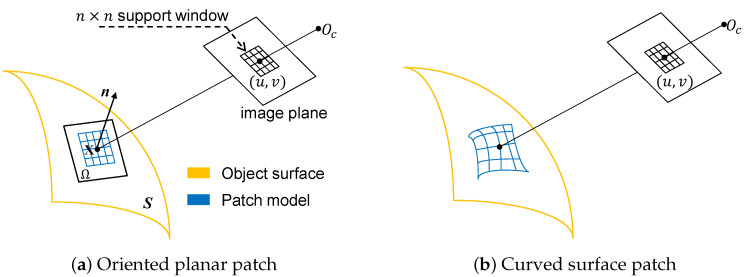
Patch parametrization methods. (**a**) shows the classical planar patch model that assigns a slant plane to the corresponding pixel location, and in (**b**) a smooth curved surface without geometric assumptions is utilized to model the 3D geometry visible in the pixel window.

High-quality dense modeling based on multi-view stereo evidence is the final step of the proposed reconstruction pipeline. Since the static stereo vision in our setup can provide rough depth data for each view, we thereby concentrate on enhancing the quality of depth maps, despite the fact that in MVS community other efficient methods [13] exist. Our proposed MVS method is capable of delivering detailed reconstruction for fine structures while at the same time inferring plausible geometry for weakly-textured regions. It consists mainly of two steps: **local patch-based optimization** and **parallel patch-wise refinement**. As a surface patch is the basic unit being processed by our algorithm, the respective patch parametrization methods (see Figure 5) as well as the corresponding objective formulations utilized in different stages, are described in the following.

#### 3.3.1. Notations

For consistency in the expression, the camera pose is represented as the transformation matrix T∈ SE(3) composed of rotation R and translation t that transforms a 3D point **X** from the world frame to camera frame. The point is further projected onto an image location **x**$={\left(u,v\right)}^{T}$ through the camera projection matrix **K**. Furthermore, we use the symbol $x^{st}$ to denote the neighboring pixel of **x**, which is located at the coordinates (u+s,v+t). The intensity image function is defined as *I*, depth function as *D* and depth derivatives against image coordinates as $D_{u}$ and $D_{v}$ respectively.

#### 3.3.2. Local Patch-Based Optimization

The oriented planar patch model has been widely employed in MVS reconstruction [24,25,26], which enables fast and accurate 3D modeling especially when combined with efficient matching algorithms [27]. In this parametrization, the scene geometry visible in the pixel window centered at the pixel (u,v) is assumed to lie on the local tangent plane Ω at the corresponding surface point, represented by the 3D coordinates **X** and its normal vector n as shown in Figure 5a. Thereby, the core idea of MVS algorithms built on this planar surface assumption is to find the plane parameters that achieve the maximal aggregated photo-consistency measures across multiple views. We employs this slant plane model in the first stage of dense reconstruction to propagate and optimize raw stereo depth data.

Our implementation is built on the framework proposed by Goesele et al. [25] that formulates the parameter estimation problem as the minimization of the sum of squared differences (SSD) based on the oriented planar structure parametrized by the depth and its derivatives (surface normal can be derived from depth derivatives). In particular, we use the inverse depth parametrization along the optical axis to replace the original direct depth parametrization along the line of sight, due to the fact pointed out in [28] that the former creates an ideal planar structure whereas the latter results in a curved surface when back-projecting an affine function (i.e., non-zero derivatives) into 3D space. Another difference is that instead of the region growing in [25] the depth estimate along with its derivatives is alternately propagated along the vertical and horizontal scanlines as in [29], in order to traverse as many regions as possible. Following these definitions, the oriented plane corresponding to the local window centered at pixel $x_{i}$ in the view *i* is expressed as

(1) $$X_{i}^{st}=\underset{D\left(x_{i}^{st}\right)}{\underset{︸}{\left(D\left(x_{i}\right)+sD_{u}\left(x_{i}\right)+tD_{v}\left(x_{i}\right)\right)}}\cdot K^{-1}x_{i}^{st},$$

where $x_{i}^{st}$ denotes the pixel location in the neighborhood with (s,t) pixel offsets from the central pixel $x_{i}$, and $X_{i}^{st}$ the 3D position backprojected from the pixel $x_{i}^{st}$. The depth value at $x_{i}^{st}$ is derived from the central depth $D(x_{i})$ as well as its gradients $D_{u}\left(x_{i}\right)$ and $D_{v}\left(x_{i}\right)$ (Note that it is actually the inverse depth utilized in our implementation, but for simplicity all reciprocal symbols are omitted in the formulations). Following the SSD metric used in [25], the optimal parameter estimates $\theta :=(D,D_{u},D_{v})$ at pixel $x_{i}$ is achieved by minimizing the aggregated intensity differences over its neighboring views, i.e.,

(2) $$\theta^{*}:=\underset{\theta }{\arg\min}\sum_{j\in N_{i}}\sum_{s,t=-m}^{m}\omega \left(x_{i}^{st}\right){∥I_{i}\left(x_{i}^{st}\right)-I_{j}\left(P_{ij}\left(X_{i}^{st}\right)\right)∥}^{2}$$

with $P_{ij}\left(X_{i}^{st}\right)=KT_{j}T_{i}^{-1}X_{i}^{st}$ denoting the projected pixel location of $X_{i}^{st}$ in view *j*, and $I_{i}$ ($I_{j}$) representing the intensity image of view *i* (*j*). Furthermore, $N_{i}$ represents the candidate neighboring views selected for view *i*, $m=\frac{n-1}{2}$ the half size of support window and $\omega (x_{i}^{st})$ the bilateral weighting function that reflects the difference of the current pixel from the central pixel in terms of distance and intensity. The non-linear weighted least squares problem in Equation (2) can be efficiently solved using the Levenberg-Marquardt method [30], and a multi-threading program is implemented on the CPU to perform the optimization, where each thread traverses the pixels located in one row (column). A post-filtering process is subsequently applied to remove incorrect estimates by checking geometric consistency over neighboring depth maps and a confidence map is created by computing normalized cross correlation (NCC) scores for the current estimates, which will be exploited in the next step.

#### 3.3.3. Parallel Patch-Wise Refinement

**Figure 6:**
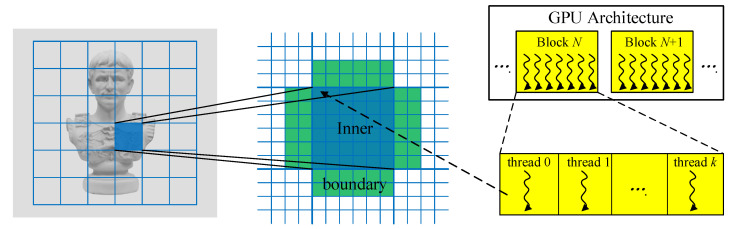
GPU-based parallel refinement. The ROI is firstly subdivided into squared patches (**left**), and all pixels in one single patch are simultaneously optimized with an additional two-pixel wide boundary for derivatives computation and regularization (**middle**). On GPU, each patch is processed by one thread block that has the same number of threads as the pixels in the patch (**right**).

Despite their high computation efficiency, *local methods* suffer from outliers and generally fail to recover smooth geometric surfaces. As an alternative, *global methods* aims to directly seek a continuous smooth model by jointly maximizing photo consistency measures and smoothness terms assuming that the scene geometry can be well modeled by smooth curved surface as shown in Figure 5b. Traditional global methods, such as variational inference [28,31,32], mostly construct their objectives over all pixels in the image domain, thus leading to scalability issue when dealing with large datasets or high resolution images. Besides, a good initial surface is critical to global optimization due to the inherent strong non-convexity of this non-linear problem. Inspired by the work in [8] originally proposed to improve depth quality using shading cues using a regular grid GPU-based Gauss-Newton solver, we implement an efficient refinement algorithm, illustrated in Figure 6, that enables parallel optimization over pixels located in sub-divided patches instead of the whole image domain, based on multi-view stereo evidence, geometric regularization terms, and depth priors. The output result from the local optimization in the first step provides the initial guess for this high-dimensional non-linear optimization problem.

Similar to [8], these sub-divided patches are iteratively refined followed the *Schwarz Alternating Procedure*, where every patch is locally optimized in one thread block and interacts with neighbor patches through boundary conditions. The depth estimates of an additional two-pixel wide boundary are required to compute depth derivatives as well as to impose constraints on the patch optimization (see Figure 6). The boundary pixel depths remain fixed during the optimization of the inner pixels. Different from the work in [8], that formulates its energy function on shading evidence in individual images and a Laplacian smoothness term, our objective is designed to incorporate stereo cues across multiple views $E_{photo}$, surface curvature-derived regularization term $E_{reg}$ as well as depth prior constraints $E_{prior}$, which is given as

(3) $$E_{total}\left(\xi \right)=\omega_{\alpha }E_{photo}\left(\xi \right)+\omega_{\beta }E_{reg}\left(\xi \right)+\omega_{\gamma }E_{prior}\left(\xi \right),$$

with $\xi ={\left[...,D\left(u-1,v\right),D\left(u,v\right),D\left(u+1,v\right),...\right]}^{T}\in R^{n^{2}}$ being the parameter vector of all depth estimates of pixels located in a n×n window. Though the original optimization problem (for the whole image domain) is partitioned into a set of independent sub-tasks defined on these sub-divided patches, the local objective (Equation (3)) for each patch still has a relatively high number of variables (e.g., the 32×32 patch used in our implementation leads to 1024 unknown parameters), making the optimization prone to problems from outlier data terms. Therefore, we employ the point-wise gradient differences proposed by Semerjian et al. [31] as our photo measure due to its better resilience to illumination variations and affine distortions compared to direct differences in raw intensity. For one single pixel $x_{i}$ located in target view *i* and visible in the neighbor view *j*, this measure across these two views is written as

(4) $$r_{ij}\left(x_{i},D\left(x_{i}\right)\right)=\nabla I_{i}\left(x_{i}\right)-\nabla I_{j}\left(\pi_{ij}\left(x_{i},D\left(x_{i}\right)\right)\right),$$

where $\pi_{ij}\left(x_{i},D\left(x_{i}\right)\right)=KT_{j}T_{i}^{-1}\left(D\left(x_{i}\right)\cdot K^{-1}x_{i}\right)$ denotes the warped pixel location of $x_{i}$ with the depth $D\left(x_{i}\right)$ in the neighbor view *j*, and *∇* the gradient operator applied on the intensity image against the image coordinates at $x_{i}$ (The Jacobian used to transform the local coordinates of the gradient vector is integrated into the gradient operator). Apart from gradient difference between the target view and its neighbors, this measure between any two neighbor views is also accumulated into the objective, as illustrated in [31,32]. Given one n×n patch centered at the pixel $x_{i}$, its photometric data term is formulated as follows

(5) $$E_{photo}\left(\xi \right)=\sum_{s,t=-m}^{m}\sum_{j,k\in N_{i}}^{j>k}Z_{j}\left(x_{i}^{st}\right)Z_{k}\left(x_{i}^{st}\right){∥r_{jk}\left(x_{i}^{st},D\left(x_{i}^{st}\right)\right)∥}^{2},$$

where $N_{i}$ is the set of neighbors of view *i* including itself, and $Z_{j}\left(x_{i}^{st}\right)$$\left(Z_{k}\left(x_{i}^{st}\right)\right)$ is a visibility indicator function that is set to 1 if the 3D point corresponding to the pixel $x_{i}^{st}$ can be observed in view *j*(k) and otherwise set to 0. Specifically, we use the input coarse depth data to set the visibility indicators for the reference view, assuming the geometric consistency between the reference view and its neighboring views [24]. With regard to the regularization term, we use the the first derivatives of surface normal vector $n={\left(n_{x},n_{y},n_{z}\right)}^{T}$ with respect to image coordinates [31]

(6) $$c\left(x_{i},\xi \right)={\left(\frac{\partial n_{x}}{\partial u},\frac{\partial n_{x}}{\partial v},\frac{\partial n_{y}}{\partial u},\frac{\partial n_{y}}{\partial v},\frac{\partial n_{z}}{\partial u},\frac{\partial n_{z}}{\partial v}\right)}^{T}\in R^{6},$$

with the components of normal vector at $x_{i}$ being computed from its depth $D(x_{i})$ as well as depth derivatives $D_{u}\left(x_{i}\right)$ and $D_{v}\left(x_{i}\right)$ approximated through central difference on neighboring depth estimates in the parameter vector ξ. This metric reflects the mean curvature of the surface without involving the scale of the 3D model due to the differentiation in image space. The total smoothness quantity is obtained by aggregating this term over all pixels in local patch

(7) $$E_{reg}\left(\xi \right)=\sum_{s,t=-m}^{m}G\left(x_{i}^{st}\right){∥c(x_{i}^{st},\xi )∥}^{2}.$$

The per-pixel weighting function $G\left(x\right)=\exp\left(-\frac{{\left(∥\nabla I(x)∥/\tau_{g}\right)}^{2}}{2\sigma_{g}^{2}}\right)$ adjusts the weight of the regularization term based on the magnitude of gradients. In addition, in order to constrain the optimization direction we also define a prior term exploiting the output result from the first stage

(8) $$E_{prior}\left(\xi \right)=\sum_{s,t=-m}^{m}\hat{C}\left(x_{i}^{st}\right){∥n\left(x_{i}^{st}\right)\cdot {\left(X_{i}^{st}-{\hat{X}}_{i}^{st}\right)}^{T}∥}^{2},$$

with

$$\left(X_{i}^{st}-{\hat{X}}_{i}^{st}\right)=\left(D\left(x_{i}^{st}\right)-\hat{D}\left(x_{i}^{st}\right)\right)\cdot K^{-1}x_{i}^{st}.$$

This measure penalizes the deviation from the initial surface by computing the point-to-plane distance, where $\hat{D}$ and $\hat{C}$ are the estimated depths as well as the corresponding confidence values (i.e., NCC score) from the local optimization. Finally, we assign different coefficients $\omega_{\alpha }$, $\omega_{\beta }$ and $\omega_{\gamma }$ in Equation (3), to different energy terms in order to balance their numeric scales as they are measured in different numeric domains.

Before running the optimization on the GPU, we first load the gradient images (for the target view and neighbor views), the initial depth map and confidence map of the target view as well as the co-visibility map derived by checking the geometric consistency between initial depths to the global memory of GPU. Afterwards, a set of thread blocks are launched to execute the patch-wise optimization. Except for the read-write operations before and after optimization that needs to exchange data with global memory, the shared memory along with registers in the thread block are exploited for most computations, which is much faster than global memory access. In the specific implementation we perform 15 Schwarz iterations in total, and in each iteration all the sub-divided patches are optimized using the Gauss-Newton solver on the GPU. And the optimization process for one patch includes two main steps:

(1) linearizing the non-linear optimization problem by computing the residuals and Jacobians for Equation (3) using one thread per pixel, resulting in a linear system;
(2) Solving this linear system in parallel using the conjugate gradient (CG) method that can be efficiently implemented within one thread block (We refer the interested readers to the paper [8] for more details about the implementation of CG solver on GPU).

In the end, a depth filtering process [24,33] is applied to the estimated depth maps to remove the potential erroneous estimates. For each view, its depth estimates are converted to 3D points that are further projected to the image planes of its neighboring view. If the relative difference between the projected depth and the depth estimate in the projected coordinate of the neighbor view is lower than 0.015, we consider these two estimates are consistent. The depth estimate that has no less than two consistent neighboring estimates is retained, otherwise it is removed.

## 4. Experiments and Results

In this section we test the proposed parallel MVS algorithm on benchmark data as well our own synthetic dataset, and afterwards demonstrate the complete 3D modeling pipeline (data acquisition and preprocessing, pose estimation and dense reconstruction) with the designed triple-camera measurement setup in a general outdoor scenario. The reconstruction results are compared with three other representative libraries in MVS community: OpenMVS [34] based on a multi-view variant of Patchmatch stereo [27], OpenMVE [35] built on slanted plane constrained least squares matching [25], and SMVS [32] that contains the implementation of the variational approach [31].

Our work (as well as SMVS) are implemented within the framework of OpenMVE, and adopt the same basic data structures and file formats as OpenMVE. For the convenience and consistency in subsequent comparison, we transplant the original implementation of OpenMVS (only the core dense reconstruction module) to the framework of OpenMVE as well, and no obvious difference in reconstruction quality is observed during our simulations. Another benefit for such a transplantation is that some parameters (e.g., NCC threshold and number of neighbor views) related to the reconstruction process can be unified and the same post procedures such as depthmap filtering and meshing, are applied for these evaluated approaches. Note that our parallel refinement algorithm is executed on an input coarse geometry, and for the fairness an interface function is programmed for other three methods to seed the reconstruction process from an external input 3D model rather than their respective original initialization. All evaluated libraries are executed on a PC with an Intel Core i5-6600K CPU with 3.5 GHz (16 GB Ram) and an Nvidia GeForce GTX 1080 GPU. We empirically select the parameters in the implementation as follows: $\omega_{\alpha }=1$, $\omega_{\beta }=0.025$ and $\omega_{\gamma }=0.01$. The squared patch size is set to 32×32, and the number of neighbor views to 4.

### 4.1. Benchmark Data

**Figure 7:**
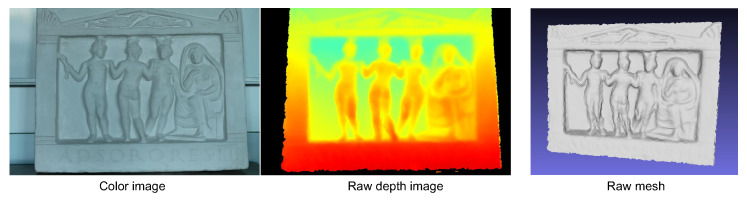
The *Relief* dataset. From left to right are the color and raw depth images for the fourth view, as well as the initial reconstruction from [36].

**Figure 8:**
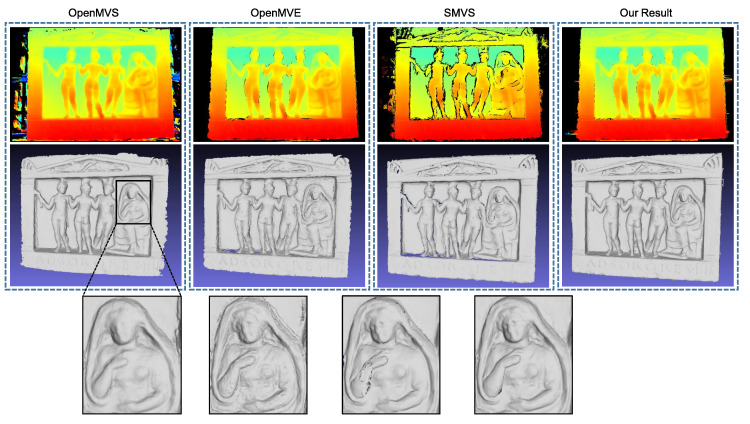
Results on the *Relief* dataset. From left to right are the output results from OpenMVS, OpenMVE, SMVS and the proposed method. The top row displays the optimized depth map for the fourth view, and the middle the reconstructed 3D mesh models and in the bottom are zoomed-in views.

We start with the *Relief* dataset provided by Zollhoefer et al. [36], where camera poses and a coarse 3D model along with the aligned depth maps are available, as shown in Figure 7. Taking this coarse model as an initial input, Figure 8 presents the output results of the evaluated methods. For our implementation, the local optimization in the first stage is skipped since a complete and clean mesh is already provided by this dataset. We can observe that the reconstructed surface by OpenMVS recovers less fine structures than its counterparts despite of its high efficiency inherited from the randomized Patchmatch algorithm, and OpenMVE produces the most detailed geometry but in the meantime generates more noise and artifacts. Due to the use of the global regularization term, SMVS (we disable the shading term in its energy function) undoubtedly obtains the smoothest 3D shape with some fine details being blurred. In contrast, our approach preserves the detailed structures of the object while suppressing the noise in textureless regions effectively. It is worth noting that our parallel algorithm adopts the basically same photo measure and regularization term (as well as the weighting factor) as SMVS. The differences in results mainly come from the utilized optimization strategies and surface representation methods.

**Figure 9:**
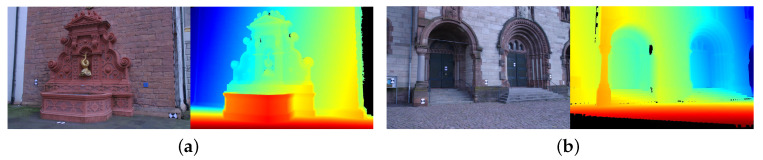
Sample color images and their ground truth depthmaps. (**a**) shows the images from *Fountain-P11* and in (**b**) are samples from *Herz-Jesu-P8*.

**Figure 10:**
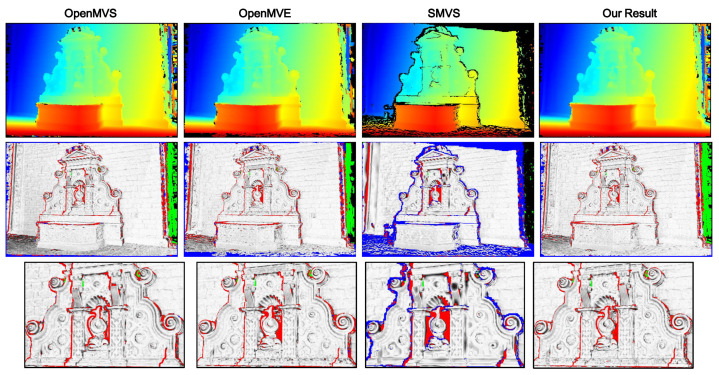
Colorized depth maps and visualized (relative) depth errors defined as $e_{rel}=e_{abs}/GT$ (GT denotes the ground truth depth) on *Fountain-P11*. From left to right: the top row displays the estimated depth map from MVS, MVE, SMVS and our method respectively; the middle and bottom rows demonstrate the per-pixel relative depth errors with blue pixels encoding missing depth data, green representing extra estimated depth values and red denoting those pixels with $e_{rel}$ larger than 0.01. The pixels with $e_{rel}$ below 0.01 are linearly converted to gray values, where the integer 255 (highest brightness) corresponds the zero deviation.

**Figure 11:**
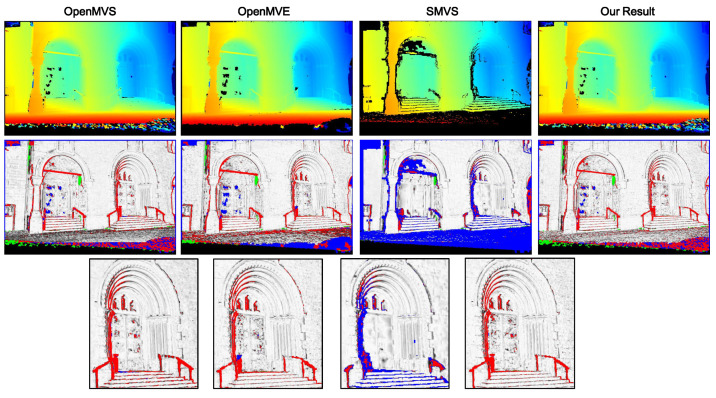
Colorized depth maps and visualized (relative) depth errors on *Herz-Jesu-P11*.

To present quantitative analysis, two outdoor benchmark datasets [37] with ground-truth models (*Fountain-P11* and *Herz-Jesu-P8*) are used. For computation efficiency and memory issue, the original high-resolution images are downsampled by half to 1536 × 1024 for dense reconstruction. Figure 9 shows for each dataset a sample image as well as the corresponding ground truth depthmap (colorized) rendered from its reference model. Following the evaluation protocol adopted in [29,38], we list the statistics of absolute depth errors in Table 2, and in addition visualize the estimated depth map along with their relative depth errors like [24] in Figure 10 and Figure 11. Although both datasets provide much texture and structure evidence for traditional stereo reconstruction, our approach still yields relatively better results in terms of accuracy and completeness. The 3D model from OpenMVS has similar completeness to ours, and OpenMVE approaches our method in reconstruction accuracy (especially for fine structures). However, SMVS unexpectedly performs much worse than others due to large amounts of missing depth estimates, which might result from its view selection strategy and final boundary cutting algorithm. We take the coarse model reconstructed at a downscaled resolution (768 × 512) using OpenMVS as an initialization for all evaluated methods.

### 4.2. Synthetic Data

**Figure 12:**
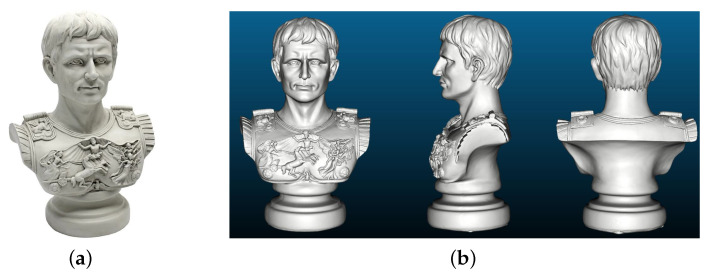
The *Caesar* sculpture (**a**) and its ground truth model (**b**). This sculpture is made of synthetic resin with a dimension of 16.5 × 26.7 × 45.7 cm, and the ground truth (reference) model is obtained using a fringe projection based industrial 3D scanner GOM-ATOS-CORE.

**Figure 13:**
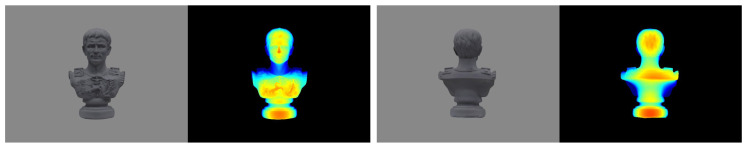
Sample synthetic color and (colorized) depth images of *Caesar* sculpture. We set in total 24 cameras poses along a circular trajectory and for each pose one aligned RGB-D images pair was rendered.

Next one synthetic dataset is created from a *Caesar* sculpture along with its reference geometry (as shown in Figure 12) to simulate the actual captured images using our measurement setup. We firstly texture this ground truth model using MeshLab [39] and then import the textured model into Blender [40] to generate aligned RGB-D image pairs for a set of virtual camera poses that are evenly distributed along a circle path at a distance of about 2 m from the model. The virtual camera intrinsic parameters are set to the same as that of the color camera in our setup, and Figure 13 displays the samples of rendered image pairs. Note that this object has many fine structures in the front side while in its back and bottom are weakly-textured curved surfaces, thereby allowing for a comprehensive comparison of different reconstruction algorithms. The reference model and the rendered ground truth depthmaps are exploited to quantify the deviations of the reconstructed models as well as the depth errors of the individual views.

**Figure 14:**
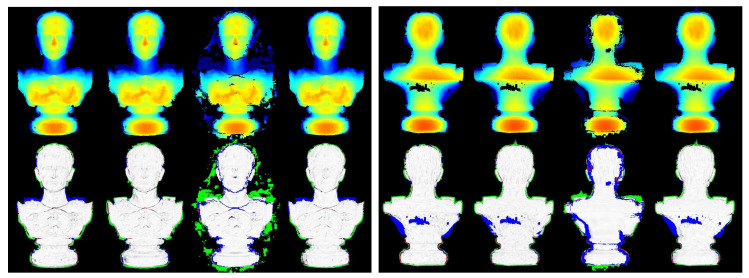
Colorized depth maps and visualized (relative) depth errors for the front and back sample views. For each view, from left to right are the estimated depth maps (**top**) and the relative depth errors (**bottom**) from MVS, MVE, SMVS and our approach respectively.

**Figure 15:**
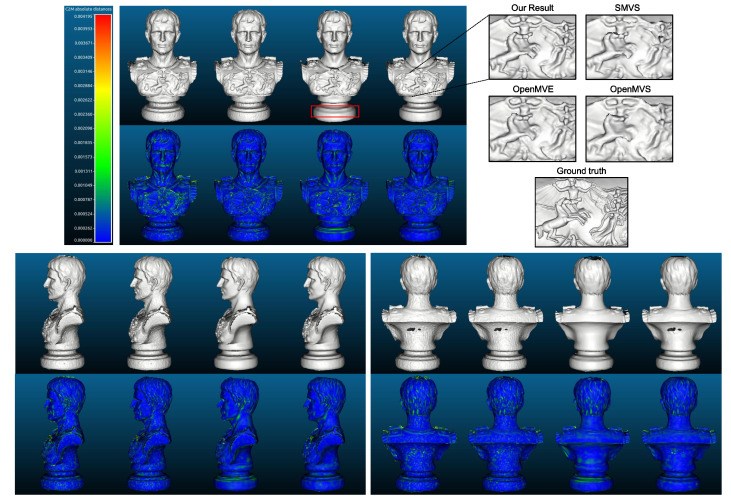
Snapshots of reconstructed models and deviation maps (meter) to the reference model from the front, side and back views. For each view, from left to right are the results of MVS, MVE, SMVS and our approach respectively. For fair comparison, the FSSR algorithm [41] integrated in MVE environment is exploited to extract the surface from dense point clouds, for all evaluated methods.

**Figure 16:**
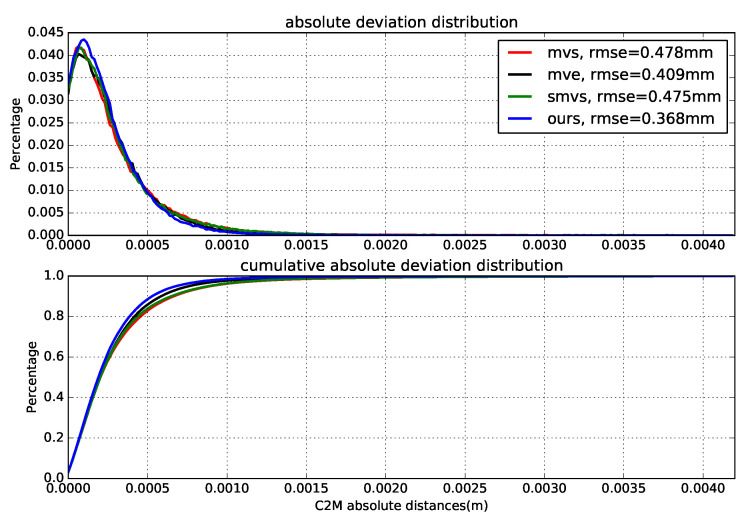
Normalized model deviation histograms and the cumulative distributions. The C2M (cloud to mesh) deviation histograms are output from CloudCompare software [42], and the respective root-mean-square error (rmse) is given in the legend.

Figure 14 demonstrates the estimated depth maps and the corresponding (relative) depth error maps for two sample views, and Table 3 presents the results for absolute depth errors. In addition, the final 3D models along with the deviations to the reference model (by computing the cloud to mesh distance) are given in Figure 15, and the normalized deviation histograms as well as the cumulative distributions are plotted in Figure 16. These results shows similar trends to that on benchmark data, where local approaches (MVS and MVE) both suffer from surface noise but MVE reveals more details (such as facial structures and relief in the front). SMVS delivers the most visually pleasant result especially for the homogeneous curved surface in the back but in the meantime leads to apparent geometry distortions (see small step marked in red rectangle of Figure 15) due to its pursuit for global smoothness. The proposed method outperforms others in overall performance when taking accuracy, completeness and noise reduction into account.

### 4.3. Experiments

**Figure 17:**
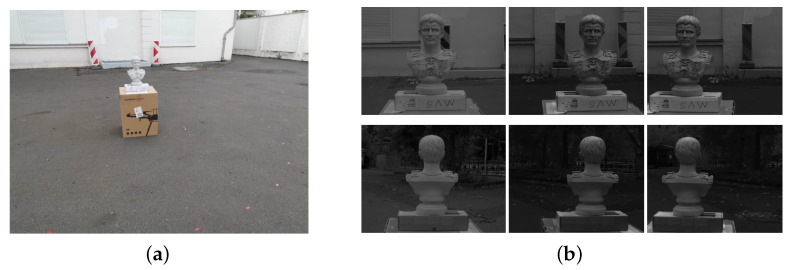
(**a**) is our experiment ground and (**b**) shows sampled triple-image pairs captured from the front (top) and back view (bottom), where from left to right are respectively the color image, the left and the right stereo images. The projected random pattern can be observed from stereo image pairs.

The experiment is conducted in the outdoor parking lot of our institute, still with this *Caesar* sculpture as the measurement object (see Figure 17a). As illustrated in Section 2, two operators are required for the image acquisition process, where one moves the measurement setup around the object by hand and the other controls the cameras from a laptop to synchronously capture images. In order to achieve 360-degree modeling, a circular trajectory with a radius of approximately 2 m centered at the measurement object is followed during the acquisition and images are evenly captured along this circle with the help of the marks on the ground. A short exposure setting is used (10 ms for stereo cameras and 12 ms for the color camera) to reduce potential blur in the images. In total 63 image pairs are selected for our reconstruction task, as shown in Figure 17b.

**Figure 18:**
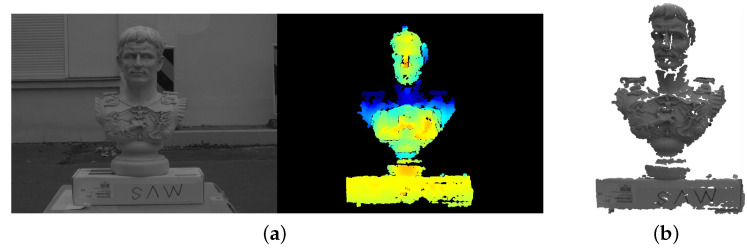
The aligned RGB-D image pair (**a**) after preprocessing as well as the generated colorized point cloud (**b**) from this pair. This point cloud is utilized by an ICP registration process for a coarse alignment, which is then be refined using the RANSAC PnP tracking module implemented in [22] that takes the RGB-D pair as input.

**Figure 19:**
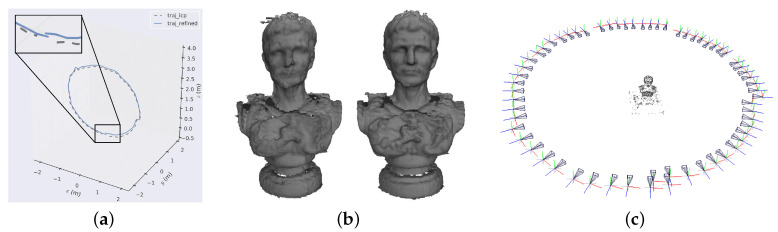
(**a**) shows the estimated camera trajectories before and after refinement, as well as the closeup view of the start and end segments. (**b**) demonstrates 3D models through volumetric integration of depth images, based on ICP-estimated poses (left) and refined poses (right) respectively. The reconstructed sparse point cloud along with the refined cameras is visualized in (**c**).

Following the detailed pipeline in Section 3, we firstly convert raw triple images into aligned RGB-D pairs (showed in Figure 18) with the calibrated setup parameters. Afterwards, multiway-ICP in combination with RANSAC PnP approach is utilized for metric-scale, accurate pose estimation, followed by loop-based pose graph optimization and global bundle adjustment [22]. Even though ground truth poses are unavailable due to the lack of external high-precision positioning device (e.g., laser tracker), apparent improvement in pose estimation accuracy can be qualitatively observed from the closure of trajectories as well as the extracted 3D meshes (see Figure 19). Since images are captured sequentially along a closed path (approx. a circle) that means cameras finally return to the starting point, the accumulated drift of the ICP approach (no overlap between the start and end segments) is displayed in the closeup view of Figure 19a (black dashed line), which is corrected after refinement (blue solid line). Figure 15b demonstrates 3D models by integrating depth images using a volumetric method [43] based on camera poses before and after refinement, where severe geometric distortions occurred due to poor accuracy of the ICP estimation before refinement.

**Figure 20:**
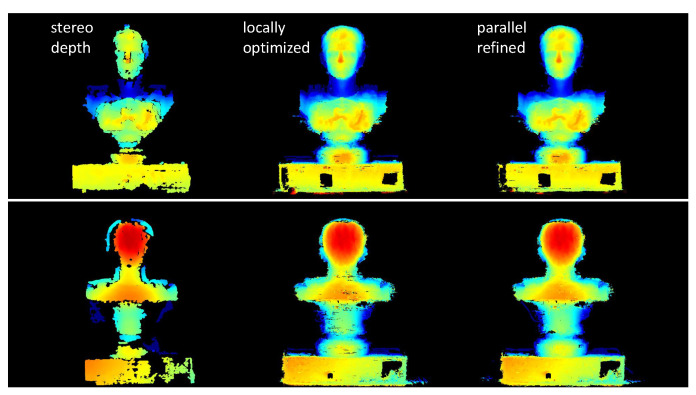
Evolution of the depth maps for the sampled front (**top**) and back (**bottom**) views. From left to right are raw depth maps from static stereo, locally optimized and parallel refined results. We can see that after local optimization process raw stereo depths are well propagated and optimized (in particular for structure-rich regions), and artifacts are filtered through geometric consistency and the NCC-based confidence check. The subsequent parallel refinement effectively suppresses noise inherited from the local optimization while preserving and sharpening fine structures of the object.

**Figure 21:**
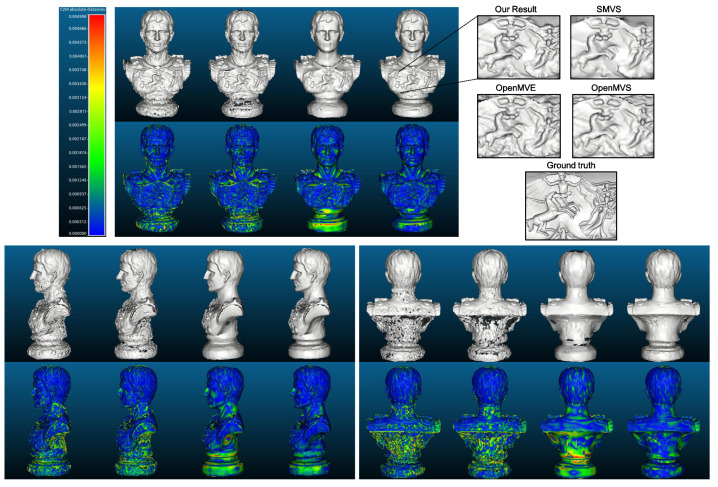
Snapshots of reconstructed models and deviation maps (meter) to the reference model from the front, side and back views. For each view, from left to right are the results of MVS, MVE, SMVS and our approach respectively. It is clear that MVS and MVE generate very uneven and noisy geometry, while SMVS based on global optimization produces a smooth but distorted surface.

**Figure 22:**
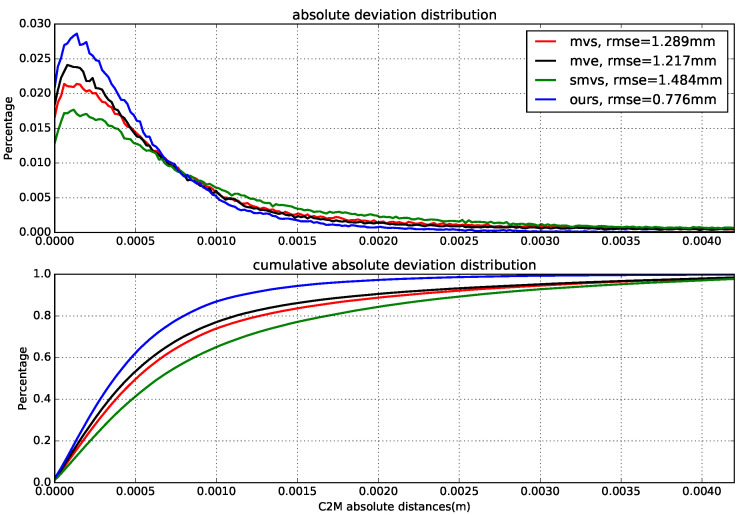
Normalized model deviation histograms and the cumulative distributions.

**Figure 23:**
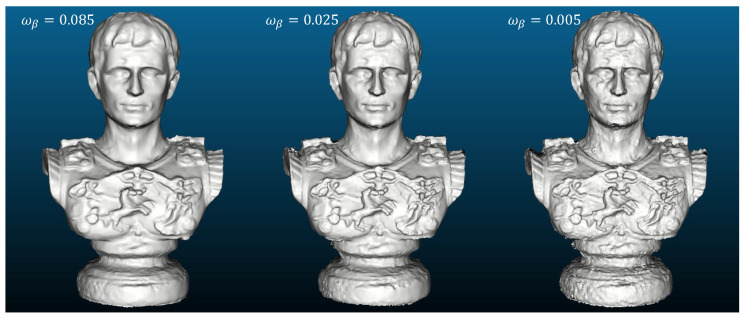
Snapshots of reconstructed models using different regularization weights. From left to right are high (0.085), medium (0.025, used in the experiment) and low (0.005) factors.

In the dense reconstruction stage, the inverse-depth parametrized local optimization (Section 3.3.1) is first executed on stereo depth maps for propagation, refinement and outlier removal. The output result is then exploited as the initial geometry for parallel patchwise refinement (Section 3.3.2) to generate detailed and smooth models. The evolution of the depth maps is illustrated in Figure 20. We present the final reconstructed models of all evaluated methods in Figure 21, which indicates that traditional local (MVS, MVE) and global (SMVS) methods both suffer for this task due to degradation in image quality (compared to synthetic data). Figure 22 gives the quantitative statistics of model deviations to the ground truth. Besides, the effect of regularization weight (i.e., $\omega_{\beta }$ in Equation (3)) in reconstruction is qualitatively investigated in Figure 23 that shows models reconstructed using different factors. Although we can see the exchange between details and smoothness with this weighting coefficient varying from high to low, the 3D shape of the object is basically maintained (no large geometric distortions like in SMVS) and the reconstruction remains mostly complete (no severe outliers like in MVS and MVE), reflecting the stability of our methods for different parameter settings to some degree. Note that this investigated variable $\omega_{\beta }$ is the factor for total regularization energy accumulated in one single patch, and for each pixel in this patch there exists an adaptive, gradient-based weighting coefficient (see Equation (7)) for its respective smoothness cost.

## 5. Discussion

The presented results from quantitative and qualitative evaluations on benchmark and synthetic datasets demonstrates that given a coarse geometry our proposed MVS reconstruction method achieves better overall performance than other representative counterparts (including local and global methods), in terms of accuracy, completeness and smoothness. The reconstructed models of these evaluated methods agree with their respective characteristics well: Local approaches produce noisy surfaces and outliers in weakly-textured regions as parameters of each pixel are independently estimated. On the other hand, global methods are capable of delivering continuous and smooth geometry by introducing regularization terms but easily get stuck in local optima (e.g., incorrect 3D shape), due to their large parameter vectors and the strong non-convexity of the underlying optimization problem. Besides, the selection of the regularization weight is usually tricky for such global methods to reach a good balance between detail recovery and surface smoothness. In contrast, our approach divides the original optimization problem (i.e., depth estimation for an individual view) into a set of sub-problems defined over subdivided square patches in the image domain, thereby reducing the complexity and parameter dimension of sub-tasks effectively and facilitating the parallel implementation of the Gauss-Newton solver on a GPU. Although this optimization strategy is theoretically sub-optimal, impressive results can still be achieved after a small number of iterations, because of its better convergence characteristics compared to conventional global optimization, as well as the usage of additional depth prior constraints.

Our experiment verifies the applicability of the designed triple-camera system to 3D digitization of real-world objects in general outdoor scenarios, in combination with the proposed reconstruction pipeline. Although active measurement techniques have been developed for several decades, efficient and high-quality modeling still remains a challenging task. The recently popular RGB-D sensors, like Kinect and RealSense cameras, suffer from the interference of ambient lighting in depth measurements and require a very short acquisition range to obtain sufficient spatial resolution for the object in color images due to their large FOV settings, which is not always available in practical cases because of the equipped platform or the location of targets. And the industrial optical scanning systems (e.g., the GOM ATOS CORE and ARAMIS series) are mostly deployed in very high-end indoor applications. From the experiment it can be seen that despite of the use of the wide stereo baseline and active projection, raw stereo depth data from single acquisition still has a relatively poor quality due to degradation in image quality and long working distance. Therefore, the camera poses estimated by running ICP on stereo depth data show apparent misalignment that needs to be mitigated using the RANSAC PnP approach, and the local optimization (Section 3.3.1) should first be performed on raw depth maps to improve the quality of initial surface, before executing our novel refinement process on the GPU (Section 3.3.2).

There are a few limitations for our system. On the hardware side, although our projector provides a much higher output power than those integrated projectors in commodity sensors, it is still not suitable for applications operated in strong environment lighting conditions (e.g., in direct sunshine) that would make our active system degrade into a purely passive stereo setup. On the software side, a good initial geometry still plays an important role in our proposed algorithms, and in sharp depth discontinuities distortions and artifacts might arise and cannot be easily fixed during the optimization.

**Figure 24:**
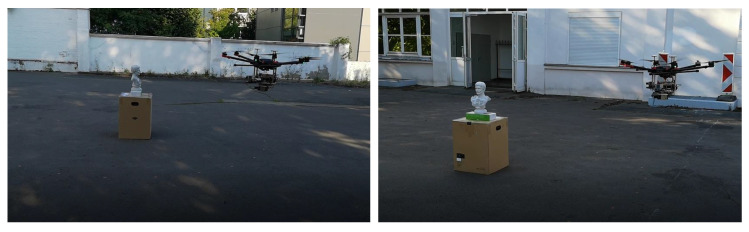
Sample images of the first prototype of our aerial measurement system. So far we have tried simple flight experiments with a hexacopter (DJI Matrice 600 PRO), and several key problems still remain to be solved, such as smooth path planning and electromagnetic interference of on-board sensors on GPS signals.

Since our measurement setup is designed to be equipped on a flight platform for aerial data acquisition, the hand-held experiment is the first step to test the performance of the proposed system for outdoor reconstruction, and in future work we would mount the setup on a hexacopter and capture images in a aerial manner, as shown in Figure 24. For this purpose, mechanical compatibility between the measurement setup and the hexacopter need to be carefully tested, and the related electronic issues remain to be addressed. Afterwards, the same reconstruction routine will be applied on the aerial dataset and then the results will be compared with that from grounded or hand-held data, from which we could investigate the influence of the flight platform on data quality and modeling accuracy (such as vibration of the aircraft) and make further modifications in hardware design and software development. In the future we hope to present an aerial measurement system along with the corresponding software package, which is capable of undertaking high-quality outdoor modeling tasks.

## 6. Conclusions

In this paper we present a triple-camera measurement system as well as the specially designed MVS reconstruction routine, able to achieve detailed and complete modeling even in an uncontrolled outdoor environment. Our hardware is composed mainly of an active stereo setup for depth acquisition, one color camera equipped with an optical filter for texture capture and a mini-PC that communicates with a client laptop and controls the data acquisition process. In order to yield high-quality results, a novel reconstruction routine is proposed to fully exploit the aligned depth and color frames converted from raw image pairs. The raw depth data from static stereo is first utilized for obtaining metric-scale camera poses and then taken as input by our MVS reconstruction modules. We evaluate our approach on benchmark and synthetic data comprehensively. The results demonstrate that the proposed parallel refinement algorithm performs better compared to other conventional methods in terms of noise reduction and surface accuracy. Finally, an outdoor reconstruction experiment is conducted using our setup in a hand-held manner, detailing the proposed pipeline from data acquisition to pose estimation to dense reconstruction, and proving that our system can be applicable to practical modeling tasks. Despite of some limitations as discussed in Section 5, we believe that the proposed work in this paper makes a meaningful attempt on 3D dense modeling in outdoor environments, and could provide some experience for further research in this field.

## Figures and Tables

**Table 1:** Intrinsic and extrinsic parameters of triple cameras. The translation vector is given in millimeters (mm) and the rotation matrix presented in Euler angles (degrees) defined with the XYZ convention. Since the depth (disparity) map is computed in the rectified coordinate of left camera, the relative transform between left and color cameras is required to assign stereo depths to pixels in color images, thus creating pixel-wise aligned RGB-D pairs used by subsequent modules.

Camera	$\left(f_{x},f_{y}\right)$	$\left(c_{x},c_{y}\right)$
Left	(3279.27, 3287.88)	(651.93, 500.69)
Right	(3324.18, 3330.10)	(659.09, 486.60)
Color	(3513.29, 3520.75)	(755.06, 597.56)
**CameraPair**	$\left(t_{x},t_{y},t_{z}\right)$	(ϕ,θ,ψ)
Left-Right	(−551.75, −2.99, 70.25)	(−0.4911, 10.9834, 0.4337)
Left-Color	(−268.77, −23.46, 39.04)	(0.4932, 5.6932, −0.3355)

**Table 2:** Ratio of pixels with absolute errors less than 2 cm and 10 cm on *Fountain-P11* and *Herz-Jesu-P8*.

		MVS	MVE	SMVS	Ours
*Fountain-P11*	2 cm	0.796	0.793	0.716	**0.825**
	10 cm	0.935	0.908	0.812	**0.941**
*HerzJesu-P8*	2 cm	0.642	0.653	0.622	**0.681**
	10 cm	0.876	0.868	0.756	**0.887**

**Table 3:** Ratio of pixels with absolute errors less than 1 mm and 5 mm on synthetic *Caesar*.

		MVS	MVE	SMVS	Ours
*Caesar-24*	1 mm	0.595	0.605	0.620	**0.662**
	5 mm	0.914	0.925	0.868	**0.931**

## Abbreviations

The following abbreviations are used in this manuscript:

3D	three-dimensional
2D	two-dimensional
MVS	multi-view stereo
ICP	iterative closest point
RANSAC	random sample consensus
PnP	perspective-n-point
GPU	graphics processing unit
FOV	field of view
TSDF	truncated signed distance function
TCP	transmission control protocol
ROI	region of interest
DMD	digital micromirror device
DC	direct current
SDK	software development kit
SLAM	simultaneous localization and mapping
SSD	sum of squared differences
NCC	normalized cross correlation
CPU	central processing unit
CG	conjugate gradient
